# High prevalence of *bla*_CTX-M-55_-carrying *Escherichia coli* in both ceftiofur-use and non-use pig farms

**DOI:** 10.1128/aem.02525-24

**Published:** 2025-07-15

**Authors:** Ji-In Kim, Bo-Youn Moon, Md. Sekendar Ali, Hee-Seung Kang, Ji-Hyun Choi, Jae-Myung Kim, Seung-Chun Park, Suk-Kyung Lim

**Affiliations:** 1 Bacterial Disease Division, Animal and Plant Quarantine Agency 65359, Gimcheon-si, Republic of Korea; 2 Laboratory of Veterinary Pharmacokinetics and Pharmacodynamics, Institute for Veterinary Biomedical Science, College of Veterinary Medicine, Kyungpook National University 579998, Daegu, Republic of Korea; Washington University in St. Louis, St. Louis, Missouri, USA

**Keywords:** *Escherichia coli*, pigs, ceftiofur resistance, extended-spectrum β-lactamase, transposable elements, sequence type

## Abstract

The emergence of third-generation cephalosporin-resistant bacteria poses a significant human health hazard worldwide. We aimed to investigate the prevalence of extended-spectrum β-lactamase (ESBL)/AmpC β-lactamase-producing third-generation cephalosporin-resistant *Escherichia coli* in ceftiofur-use and non-use pig farms. A total of 585 *E. coli* isolates were collected from pig feces (*n* = 354) and farm environments (*n* = 231) across various farms between 2022 and 2023 in South Korea. Among them, 61% of *E. coli* from pigs and 22.9% from the environment were resistant to ceftiofur, with significantly higher rates in ceftiofur-use farms. The most common ESBL gene detected was *bla*_CTX-M-55_ (59.9%), followed by *bla*_CTX-M-15_ (12.3%) and *bla*_CTX-M-14_ (7.4%). Resistance to non-beta-lactam antimicrobials varied, with *bla*_CTX-M-55_-carrying *E. coli* showing high resistance. Gene transfer via conjugation was observed, particularly for *bla*_CTX-M-1_ (85.7%) and *bla*_CTX-M-55_ (46.6%). Of note, seven isolates co-harboring *mcr-1* and *bla*_CTX-M-55_ were found in pigs. Replicon type IncFIB (85.3%) was mainly found in *bla*_CTX-M-55_-carrying plasmids and IncI1 (91.7%) in *bla*_CTX-M-1_. The most frequently observed sequence types were ST457, ST410, ST101, ST75, and ST10, which comprised 38.4% of the isolates. The transposable elements IS*26*-IS*Ecp1-bla*_CTX-M-55_-*orf477* (76%), IS*Ecp1-bla*_CTX-M-55_-*orf477* (22.7%), *bla*_CTX-M-65_-IS*903* (50%), and *bla*_CTX-M-14_-IS*903* (100%) were predominantly detected. This study implies that ceftiofur use, clonal, and horizontal transfer might cause a high prevalence of ESBLs in pig farms. Thus, these findings emphasize the importance of prudent antimicrobial use and biosecurity measures to combat antimicrobial resistance.

**IMPORTANCE:**

Antimicrobial resistance in bacteria is a growing concern for both humans and animals. Pigs and their farm environment can carry and spread antimicrobial-resistant bacteria. Moreover, these resistant bacteria can be transmitted to humans and cause complications in treating infections. In this investigation, we present evidence for the widespread occurrence of third-generation cephalosporin-resistant extended-spectrum β-lactamase (ESBL)/AmpC β-lactamase (AmpC) genes in *Escherichia coli* isolates in ceftiofur-use pigs, which were clonally and horizontally transferred and detected significantly in ceftiofur non-use pigs and their farm environment. Furthermore, we observed different transposable elements, sequence types, and replicon types of *E. coli* that potentially participate in disseminating antimicrobial resistance. Our study will contribute to a better understanding of the emergence and dissemination of ceftiofur-resistant *E. coli* and develop a strategy for preventing its spreading to humans and other animals.

## INTRODUCTION

The third-generation cephalosporins are among the high-priority and critically important antimicrobials used to treat bacterial infections in both humans and veterinary practice (1). Resistance to third-generation cephalosporin is mostly caused by producing extended-spectrum β-lactamase (ESBL)/AmpC β-lactamase (AmpC) enzymes that can deactivate the β-lactam ring, rendering them ineffective (2). *Escherichia coli* capable of producing ESBL/AmpC β-lactamase are a significant concern due to their negative impact on humans and other animals.

Research conducted on farm animals demonstrated that factors such as antimicrobial exposure and farm environment can affect the levels and patterns of antimicrobial resistance in *E. coli* (3, 4). The excessive use and abuse of β-lactam antimicrobials, including third-generation cephalosporin, in veterinary practice settings has led to the rise of ESBL/AmpC-producing *E. coli* in animal husbandry (5). Numerous studies have reported an increase in the prevalence of ESBL/AmpC-harboring *E. coli* in food-producing animals in several countries in Europe (6, 7), the USA (8), Africa (9, 10), and Asia (11, 12). Especially pigs are regarded as one of the main causes of the rising prevalence of third-generation cephalosporin-resistant *E. coli* in food animals (13). This epidemiological tendency can be related to the prolonged and widespread use of third-generation cephalosporins, including ceftiofur, in swine production (14). In contrast, studies have been published to describe the prevalence of ESBL-harboring *E. coli* in cephalosporin non-use pigs, which might be due to the use of other β-lactam antimicrobials, including carbapenems, penicillin, or monobactams (15).

The potential for ESBL/AmpC-carrying *E. coli* to spread among the pigs and farm environment has been found in previous investigations (16, 17). Different routes for transmission of resistant bacteria have been proposed, including direct contact among the pigs in the herd and contact of discharged manure with the farm environment, such as the floor, fence, and ventilation (18). These resistant bacteria in the farm environment can persist for an extended period in the existing farm settings and can be a source of infections (19). Therefore, previous investigations on the prevalence of antimicrobial resistance have been conducted not only in animals but also in their surrounding environment to effectively measure and decrease the potential spread of antimicrobial resistance in animals and humans.

The occurrence of *E. coli* harboring ESBL/AmpC β-lactamase genes in pigs in Korea has been investigated in several studies (17, 20–22); however, information on ceftiofur use or non-use, prevalence of various age groups, and their farm environment is still scarce. Thus, the current investigation aimed to determine the prevalence and molecular characteristics of ESBL/AmpC-producing *E. coli* in ceftiofur-use and non-use farms.

## RESULTS

### Prevalence of ceftiofur-resistant *E. coli* at sample level

Overall, ceftiofur-resistant *E. coli* was isolated in 46% (269/585) of samples, comprising 61% (216/354) from pigs and 22.9% (53/231) from the farm environment (Table 1). Both in the pig and environment, the prevalence of ceftiofur resistance in ceftiofur-use farms was higher than in ceftiofur-non-use farms. In particular, finisher, sow, floor, and ventilation samples were significantly higher in ceftiofur-use farms (*P* < 0.05). In addition, prevalence was different by age group. Ceftiofur-resistant *E. coli* isolates were obtained from weaner piglets (71.1%), followed by growers (61.8%), finishers (55.3%), and sows (55.6%). Interestingly, ceftiofur resistance was highest in the finisher group of the ceftiofur-use farm (90%).

**TABLE 1:** Prevalence of ceftiofur-resistant *E. coli* isolated from pigs and their farm environment during 2022–2023 in South Korea

Source	Samples	Prevalence % (no. positive/no. tested)			*P*-value
		Ceftiofur use	Ceftiofur non-use	Total	
Pigs	Weaner	80.0 (20/25)	67.7 (44/65)	71.1 (64/90)	0.24
	Grower	64.0 (16/25)	60.9 (39/64)	61.8 (55/89)	0.78
	Finisher	90.0 (18/20)	44.6 (29/65)	55.3 (47/85)	<0.01
	Sow	76.0 (19/25)	47.7 (31/65)	55.6 (50/90)	0.01
	Subtotal	76.8 (73/95)	55.2 (143/259)	61.0 (216/354)	<0.01
Farm environment	Floor	78.9 (15/19)	40.4 (21/52)	50.7 (36/71)	<0.01
	Ventilation	42.1 (8/19)	15.4 (8/52)	22.5 (16/71)	0.01
	Feed	0 (0/19)	0 (0/52)	0 (0/71)	ND^*a*^
	Drinking water	0 (0/5)	7.7 (1/13)	5.6 (1/18)	0.52
	Subtotal	37.1 (23/62)	17.8 (30/169)	22.9 (53/231)	<0.01
Total		61.1 (96/157)	40.4 (173/428)	46.0 (269/585)	<0.01

^*a*^ ND, not defined.

In the farm environment, the prevalence of ceftiofur-resistant *E. coli* was also higher in ceftiofur-use farms than in non-use farms. The majority of the isolates were obtained from the floor (50.7%) and ventilation surface (22.5%) in both farms. However, no ceftiofur-resistant isolates were found in feeds; one isolate was detected in drinking water.

### Distribution of ESBL/AmpC β-lactamase genes

A total of eight different ESBL/AmpC β-lactamase genes were detected in the ceftiofur-resistant *E. coli* isolates (Table 2; Fig. S1). Among them, *bla*_CTX-M-55_ was predominantly found in both pigs and the environment. In pigs, *bla*_CTX-M-55_ was detected in 59.3% (128/216) of the isolates, followed by *bla*_CTX-M-15_ (14.8%, 32/216), while *bla*_CMY-2_ was identified in 11.1% (24/216) of the isolates, and *bla*_DHA-1_ was detected in 22 isolates (10.2%, 22/216). Interestingly, *bla*_CTX-M_ and *bla*_CMY_ genes co-occurred in 10 isolates (4.6%, 10/216), and *bla*_CTX-M_ and *bla*_DHA_ genes co-occurred in 22 isolates (10.2%, 22/216). In the farm environment, *bla*_CTX-M-55_ was identified in 62.3% (33/53) of the isolates, followed by *bla*_CTX-M-14_ (13.2%, 7/53) and *bla*_CTX-M-65_ (11.3%, 6/53), and *bla*_CMY-2_ was found in 7.5% (4/53) of the isolates, while *bla*_DHA-1_ was not observed in any isolates. Overall, the distribution of ESBL/AmpC β-lactamase genes was higher in ceftiofur-use than in ceftiofur non-use farms.

**TABLE 2:** Distribution of β-lactamase genes in ceftiofur-resistant *E. coli* isolated from pigs and their farm environment during 2022–2023 in South Korea^*a*^

Ceftiofur use/non-use	Farm ID	*bla* gene distribution % (no. of *bla* gene-carrying isolates)																				
		Pigs													Environment							
		No. of samples	*bla*_CTX-M-55_	*bla*_CTX-M-15_ + *bla*_DHA-1_	*bla*_CMY-2_	*bla*_CTX-M-65_	*bla*_CTX-M-1_	*bla*_CTX-M-14_	*bla*_CTX-M-15_	*bla*_CTX-M-55_ + *bla*_CMY-2_	*bla*_CTX-M-27_	*bla*_CTX-M-14_ + *bla*_CMY-2_	*bla*_CTX-M-1_ + *bla*_CMY-2_	Subtotal	No. of samples	*bla*_CTX-M-55_	*bla*_CTX-M-14_	*bla*_CTX-M-65_	*bla*_CMY-2_	*bla*_CTX-M-1_	*bla*_CTX-M-15_	Subtotal
Ceftiofur use	E	15	–	–	2	–	11	–	–	–	–	–	1	93.3 (14)	10	–	–	–	1	2	–	30.0 (3)
	G	20	19	–	–	–	–	–	–	–	–	–	–	95.0 (19)	13	7	–	–	–	–	–	53.8 (7)
	I	20	3	–	2	11	–	–	–	2	–	–	–	90.0 (18)	13	1	–	4	–	–	–	38.5 (5)
	L	20	4	–	2	–	–	2	–	–	–	–	–	40.0 (8)	13	2	–	–	–	–	–	15.4 (2)
	S	20	3	–	2	–	–	4	2	1	2	–	–	70.0 (14)	13	–	4	2	–	–	–	46.2 (6)
	Sub-total	95	30.5 (29)	0 (0)	8.4 (8)	11.6 (11)	11.6 (11)	6.3 (6)	2.1 (2)	3.2 (3)	2.1 (2)	0 (0)	1.1 (1)	76.8 (73)	62	16.1 (10)	6.5 (4)	9.7 (6)	1.6 (1)	3.2 (2)	0 (0)	37.1 (23)
Ceftiofur non-use	A	20	15	–	1	–	–	–	–	–	–	–	–	80.0 (16)	13	5	–	–	–	–	–	38.5 (5)
	B	20	8	–	2	–	–	–	–	–	3	–	–	65.0 (13)	13	1	–	–	1	–	–	15.4 (2)
	C	19	–	11	–	–	–	–	–	–	–	–	–	57.9 (11)	13	–	–	–	–	–	–	0 (0)
	D	20	1	11	–	–	–	–	–	–	–	–	–	60.0 (12)	13	1	–	–	–	–	–	7.7 (1)
	F	20	13	–	–	–	–	–	–	–	–	–	–	65.0 (13)	13	1	–	–	–	–	–	7.7 (1)
	H	20	15	–	–	–	–	–	–	–	–	–	–	75.0 (15)	13	2	–	–	–	–	–	15.4 (2)
	J	20	–	–	–	–	–	–	–	3	–	–	–	15.0 (3)	13	–	–	–	–	–	–	0 (0)
	M	20	8	–	–	–	–	–	–	–	–	–	–	40.0 (8)	13	–	–	–	–	–	–	0 (0)
	N	20	8	–	–	–	–	–	–	–	–	–	–	40.0 (8)	13	–	–	–	–	–	–	0 (0)
	O	20	5	–	3	–	–	–	1	–	–	–	–	45.0 (9)	13	3	–	–	1	–	1	38.5 (5)
	P	20	8	–	–	–	–	–	–	–	–	–	–	40.0 (8)	13	5	–	–	–	–	–	38.5 (5)
	Q	20	12	–	–	–	–	–	–	–	–	–	–	60.0 (12)	13	5	–	–	–	–	–	38.5 (5)
	R	20	–	–	–	1	–	4	7	–	–	3	–	75.0 (15)	13	–	3	–	1	–	–	30.8 (4)
	Sub-total	259	35.9 (93)	8.5 (22)	2.3 (6)	0.4 (1)	0 (0)	1.5 (4)	3.1 (8)	1.2 (3)	1.2 (3)	1.2 (3)	0 (0)	55.2 (143)	169	13.6 (23)	1.8 (3)	0 (0)	1.8 (3)	0 (0)	0.6 (1)	17.8 (30)
Total		354	34.5 (122)	6.2 (22)	4.0 (14)	3.4 (12)	3.1 (11)	2.8 (10)	2.8 (10)	1.7 (6)	1.4 (5)	0.8 (3)	0.3 (1)	61.0 (216)	231	14.3 (33)	3.0 (7)	2.6 (6)	1.7 (4)	0.9 (2)	0.4 (1)	22.9 (53)

^*a*^ –, not detected.

### Comparison of resistance by *bla*_CTX-M_ types

Differences in antibiotic resistance were noted according to the types of ESBL/AmpC producers and co-producers (Table 3). Among the beta-lactam antibiotics, amoxicillin/clavulanic acid showed high resistance in *bla*_CMY-2_ (100%) and co-producing *bla*_CTX-M-55_ and *bla*_DHA-1_ (100%) *E. coli*. For the fourth-generation cephalosporin, cefepime resistance was high in *bla*_CTX-M-55_ (43.2%). Of the non-beta-lactam antibiotics, resistance to chloramphenicol and tetracycline was high in all *bla*_CTX-M_ or *bla*_CMY-2_ types except the co-occurring *bla*_CTX-M-15_ and *bla*_DHA-1_ and co-producing *bla*_CTX-M-14_ and *bla*_CMY-2_. Similarly, ciprofloxacin resistance was high, ranging from approximately 20% to 60%, except for co-existent *bla*_CTX-M-15_ and *bla*_DHA-1_, *bla*_CTX-M-14_ and *bla*_CMY-2_, and *bla*_CTX-M-1_ and *bla*_CMY-2_. Additionally, colistin resistance was detected in 4.5% (*n* = 7) of *bla*_CTX-M-55_-producing *E. coli* from pigs and floor, all of which were confirmed to carry the *mcr-1* gene.

**TABLE 3:** Distribution of antimicrobial resistance in ESBL/AmpC types in ceftiofur-resistant *E. coli* isolated from pigs and their farm environment during 2022–2023 in South Korea

Antimicrobials	Antimicrobial resistance % (no. resistant)											
	*bla*_CTX-M-55_ (*n* = 155)	*bla*_CTX-M-15_ + *bla*_DHA-1_ (*n* = 22)	*bla*_CTX-M-65_ (*n* = 18)	*bla*_CMY-2_ (*n* = 18)	*bla*_CTX-M-14_ (*n* = 17)	*bla*_CTX-M-1_ (*n* = 13)	*bla*_CTX-M-15_ (*n* = 11)	*bla*_CTX-M-55_ + *bla*_CMY-2_ (*n* = 6)	*bla*_CTX-M-27_ (*n* = 5)	*bla*_CTX-M-14_ + *bla*_CMY-2_ (*n* = 3)	*bla*_CTX-M-1_ + *bla*_CMY-2_ (*n* = 1)	Total (*n* = 269)
Amoxicillin/clavulanic acid	0 (0)	100.0 (22)	0 (0)	100.0 (18)	0 (0)	0 (0)	0 (0)	100.0 (6)	0 (0)	100.0 (3)	100.0 (1)	18.6 (50)
Ampicillin	100.0 (155)	100.0 (22)	100.0 (18)	100.0 (18)	100.0 (17)	100.0 (13)	100.0 (11)	100.0 (6)	100.0 (5)	100.0 (3)	100.0 (1)	100.0 (269)
Cefepime	43.2 (67)	0 (0)	0 (0)	5.6 (1)	5.9 (1)	0 (0)	0 (0)	33.3 (2)	0 (0)	0 (0)	0 (0)	26.4 (71)
Cefotaxime	100.0 (155)	100.0 (22)	100.0 (18)	100.0 (18)	100.0 (17)	100.0 (13)	100.0 (11)	100.0 (6)	100.0 (5)	100.0 (3)	100.0 (1)	100.0 (269)
Cefoxitin	0 (0)	100.0 (22)	0 (0)	94.4 (17)	0 (0)	0 (0)	0 (0)	100.0 (6)	0 (0)	100.0 (3)	100.0 (1)	18.2 (49)
Cetazidime	43.9 (68)	4.5 (1)	0 (0)	50.0 (9)	0 (0)	0 (0)	0 (0)	100.0 (6)	0 (0)	0 (0)	100.0 (1)	31.6 (85)
Chloramphenicol	68.4 (106)	0 (0)	94.4 (17)	61.1 (11)	23.5 (4)	76.9 (10)	27.3 (3)	83.3 (5)	60.0 (3)	0 (0)	100.0 (1)	59.5 (160)
Ciprofloxacin	48.4 (75)	0 (0)	33.3 (6)	66.7 (12)	23.5 (4)	30.8 (4)	36.4 (4)	50.0 (3)	40.0 (2)	0 (0)	0 (0)	40.9 (110)
Colistin	4.5 (7)	0 (0)	0 (0)	0 (0)	0 (0)	0 (0)	0 (0)	0 (0)	0 (0)	0 (0)	0 (0)	2.6 (7)
Gentamicin	58.7 (91)	0 (0)	88.9 (16)	38.9 (7)	35.3 (6)	30.8 (4)	81.8 (9)	33.3 (2)	0 (0)	0 (0)	100.0 (1)	50.6 (136)
Nalidixic acid	61.3 (95)	0 (0)	38.9 (7)	72.2 (13)	76.5 (13)	61.5 (8)	72.7 (8)	83.3 (5)	40.0 (2)	100.0 (3)	0 (0)	57.2 (154)
Streptomycin	91.6 (142)	0 (0)	94.4 (17)	77.8 (14)	100.0 (17)	92.3 (12)	100.0 (11)	83.3 (5)	60.0 (3)	100.0 (3)	100.0 (1)	46.5 (125)
Sulfisoxazole	63.9 (99)	0 (0)	61.1 (11)	77.8 (14)	82.4 (14)	69.2 (9)	100.0 (11)	83.3 (5)	0 (0)	100.0 (3)	100.0 (1)	62.1 (167)
Tetracycline	61.3 (95)	0 (0)	94.4 (17)	94.4 (17)	88.2 (15)	100.0 (13)	36.4 (4)	100.0 (6)	80.0 (4)	100.0 (3)	100.0 (1)	65.1 (175)
Trimethoprim/sulfamethoxazole	42.6 (66)	9.1 (2)	61.1 (11)	72.2 (13)	76.5 (13)	69.2 (9)	27.3 (3)	66.7 (4)	0 (0)	100.0 (3)	100.0 (1)	46.5 (125)

### Transferability and molecular characteristics of *bla*_CTX-M_ environment

It was found that the transferability was different by *bla*_CTX-M_ types (Table 4). The majority of the *bla*_CTX-M-1_ (85.7%, 12/14) was transferred; however, the transfer of *bla*_CTX-M-55_ and *bla*_CTX-M-14_ was about 40%, and other types were less than 20%. Plasmid replicon typing results showed that IncFIB (65.4%, 68/104) was the most prevalent replicon type detected in all *bla*_CTX-M_ genes except *bla*_CTX-M-1_. The plasmid IncI1 carried the *bla*_CTX-M-1_ gene. Of note, among the 75 *bla*_CTX-M-55_ transconjugants, 64 transconjugants harbored the IncFIB plasmid. The IncI1 type was mainly detected in *bla*_CTX-M-1_ transconjugants. However, none of the isolates harboring *bla*_CMY-2_ were transferred to the recipient.

**TABLE 4:** Characterization of β-lactamase genes in ceftiofur-resistant *E. coli* isolated from pigs and their farm environment during 2022–2023 in South Korea^*a*^^,^^*b*^

Resistance genes	No. of isolates		Transferability (no. of isolates)	Replicon type	Environmental type	Transferred resistance
	Pigs (%)	Environment (%)				
*bla*_CTX-M-55_ (*n* = 161, 59.9%)	128 (59.3)	33 (62.3)	46.6 (75)	FIB (*n* = 54)	I (*n* = 49), II (*n* = 5)	CHL (*n* = 6), CHL-GEN (*n* = 7), CHL-GEN-STR (*n* = 12), CHL-STR (*n* = 8), CHL-TET (*n* = 1), CHL-TET-STR (*n* = 4), CHL-TET-STX (*n* = 7), GEN (*n* = 1), TET-GEN-STR (*n* = 1), TET-STR (*n* = 1), and TET-GM-STR-SXT (*n* = 1)
				FIB, B/O (*n* = 2)	I (*n* = 2)	CHL-TET-STR (*n* = 2)
				FIB, I1 (*n* = 6)	I (*n* = 6)	CHL-STR (*n* = 1), CHL-SXT (*n* = 1), TET-GEN-STR-SXT (*n* = 3), and CHL-GEN-STR-SXT (*n* = 1)
				FIB, I1, N (*n* = 1)	II (*n* = 1)	CHL-TET-STR (*n* = 1)
				FIB, N (*n* = 1)	II (*n* = 1)	CHL-STR (*n* = 1)
				F, Y (*n* = 1)	II (*n* = 2)	CHL-GEN (*n* = 1)
				I1 (*n* = 1)	III (*n* = 1)	CHL-GEN-STR (*n* = 1)
				Unknown (*n* = 9)	II (*n* = 9)	TET (*n* = 1)
*bla*_CTX-M-1_ (*n* = 14, 5.2%)	12 (5.6)	2 (3.8)	85.7 (12)	I1 (*n* = 7)	II (*n* = 7)	TET (*n* = 7)
				I1-N (*n* = 3)	II (*n* = 3)	TET (*n* = 3)
				I1-N-Y (*n* = 1)	II (*n* = 1)	TET (*n* = 1)
				N (*n* = 1)	II (*n* = 1)	–
*bla*_CTX-M-14_ (*n* = 20, 7.4%)	13 (6)	7 (13.2)	40 (8)	F (*n* = 1)	IV (*n* = 1)	CHL-TET-GEN-STR-SXT (*n* = 1)
				FIB, I1 (*n* = 1)	IV (*n* = 1)	CHL (*n* = 1)
				F, K2 (*n* = 1)	IV (*n* = 1)	–
				K2 (*n* = 4)	IV (*n* = 4)	–
				Unknown (*n* = 1)	IV (*n* = 1)	CHL-TET-GEN-STR-SXT (*n* = 1)
*bla*_CTX-M-15_ (*n* = 33, 12.3%)	32 (14.8)	1 (1.9)	12.1 (4)	F (*n* = 1)	II (*n* = 1)	–
				FIB, B/O (*n* = 1),	I (*n* = 1)	CHL-TET-STR (*n* = 1)
				Unknown (*n* = 2)	II (*n* = 2)	–
*bla*_CTX-M-65_ (*n* = 18, 6.7%)	12 (5.6)	6 (11.3)	22.2 (4)	FIB (*n* = 2)	I (*n* = 1), IV (*n* = 1)	TET-STR-SXT (*n* = 1)
				Unknown (*n* = 2)	IV (*n* = 1), V (*n* = 1)	STR (*n* = 1)
*bla*_CTX-M-27_ (*n* = 5, 1.9%)	5 (2.3)	0 (0)	20.0 (1)	F (*n* = 1)	IV (*n* = 1)	–
*bla*_CMY-2_ (*n* = 18, 6.7%)	14 (6.5)	4 (7.5)	0 (0)	–	–	–
Total	216	53	38.7 (104)			

^*a*^ CHL, chloramphenicol; GEN, gentamicin; STR, streptomycin; SXT, trimethoprim/sulfamethoxazole; TET, tetracycline.

^*b*^ –, not detected.

**Fig 1:**
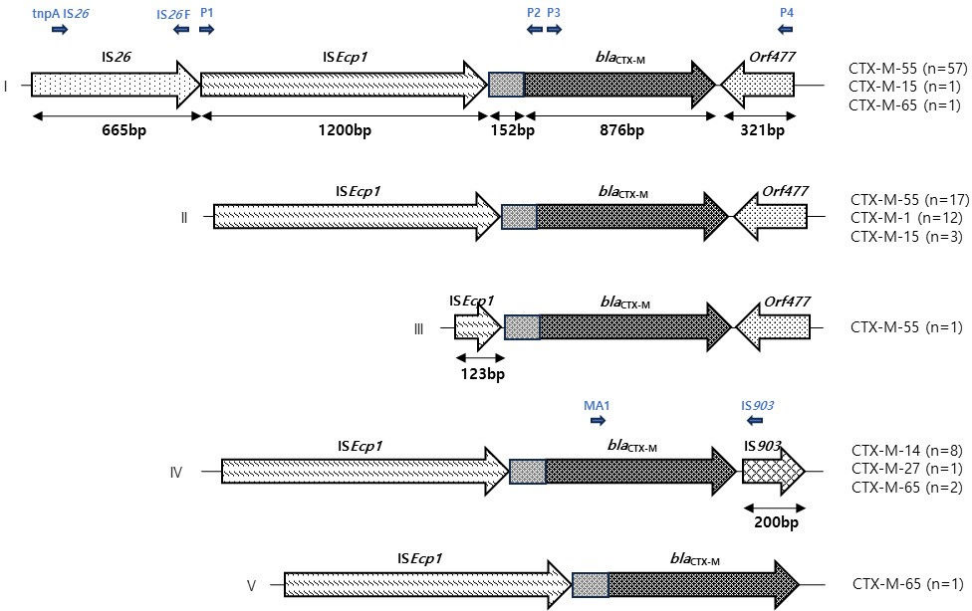
Detection of the transposable elements in the *bla*_CTX-M_ gene environment of ESBL/AmpC-carrying *E. coli*.

We identified genetic elements among 104 transconjugants, and after clustering, we obtained five types of genetic structure (I–V). These genetic types differed by *bla*_CTX-M_ and replicon types. The most common type was type I, which included complete IS*ECP1* and IS*26* upstream and *orf477* downstream (Fig. 1). Type I was predominantly found in *bla*_CTX-M-55_ (76%, 57/75), *bla*_CTX-M-15_ (25%, 1/4), and *bla*_CTX-M-65_ (25%, 1/4). Another frequently observed type was type II, which was the absence of IS*26*. This type was found in *bla*_CTX-M-55_ (22.7%, 17/75), *bla*_CTX-M-1_ (100%, 12/12), and *bla*_CTX-M-15_ (25%, 1/4). We detected IS*903* downstream of the *bla*_CTX-M_ group, *bla*_CTX-M-14_ (100%, 8/8), *bla*_CTX-M-27_ (100%, 1/1), and *bla*_CTX-M-65_ (50%, 2/4).

Moreover, different antimicrobial resistance was transferred with *bla*_CTX-M_ (Table 4). Tetracycline, chloramphenicol, and aminoglycoside resistance were transferred with *bla*_CTX-M-55_, *bla*_CTX-M-1_, *bla*_CTX-M-14_, and *bla*_CTX-M-15_ in 94 transferred isolates. Of note, all *bla*_CTX-M-55_-carrying transconjugants transferred with one more antimicrobial, tetracycline, which is commonly used in pigs.

### Pulsed-field gel electrophoresis and multi-locus sequence typing analysis

A total of 125 pulsed-field gel electrophoresis (PFGE) patterns were observed in 269 ESBL/AmpC-producing *E. coli* (Table 5). Overall, the patterns were heterogeneous, and no identical patterns were observed between different farms. However, within the same farm, identical patterns were observed between pigs, as well as between pigs and their environment.

**TABLE 5:** PFGE patterns of ESBL/AmpC-carrying *E. coli* isolated from pigs and their farm environment during 2022–2023 in South Korea^*a*^^,^^*b*^

Farm ID	*bla* types	PFGE type						
		Pigs				Environment		
		Weaning	Growing	Finishing	Sow	Floor	Ventilation fan	Water
A	*bla*_CTX-M-55_ (*n* = 20)	P065 (*n* = 3), P100 (*n* = 1), and P101 (*n* = 1)	P065 (*n* = 1), P094 (*n* = 1), P107 (*n* = 1), and P120 (*n* = 1)	P066 (*n* = 2) and P120 (*n* = 1)	P120 (*n* = 2) and P121 (*n* = 1)	P065 (*n* = 1), P066 (*n* = 1), and P122 (*n* = 1)	P123 (*n* = 1)	P107 (*n* = 1)
	*bla*_CMY-2_ (*n* = 1)	–^*c*^	–	–	P110 (*n* = 1)	–	–	–
B	*bla*_CTX-M-27_ (*n* = 3)	–	P111 (*n* = 3)	–	–	–	–	–
	*bla*_CTX-M-55_ (*n* = 9)	P014 (*n* = 5)	–	–	P092 (*n* = 1) and P095 (*n* = 2)	P014 (*n* = 1)	–	–
	*bla*_CMY-2_ (*n* = 3)	–	P007 (*n* = 1)	–	P007 (*n* = 1)	P007 (*n* = 1)	–	–
C	*bla*_CTX-M-15_ (*n* = 11)	P127 (*n* = 3)	P127 (*n* = 2)	P127 (*n* = 4)	P127 (*n* = 2)	–	–	–
D	*bla*_CTX-M-15_ (*n* = 11)	P127 (*n* = 3)	P127 (*n* = 3)	P127 (*n* = 3)	P127 (*n* = 2)	–	–	–
	*bla*_CTX-M-55_ (*n* = 2)	P084 (*n* = 1)	–	–	–	P085 (*n* = 1)	–	–
E	*bla*_CTX-M-1_ (*n* = 14)	P042 (*n* = 2) and P096 (*n* = 3)	P032 (*n* = 1), P043 (*n* = 1), and P044 (*n* = 1)	–	P008 (*n* = 1), P022 (*n* = 1), and P048 (*n* = 2)	P045 (*n* = 1) and P097 (*n* = 1)	–	–
	*bla*_CMY-2_ (*n* = 3)	–	P109 (*n* = 2)	–	–	P093 (*n* = 1)	–	–
F	*bla*_CTX-M-55_ (*n* = 14)	P038 (*n* = 5)	P038 (*n* = 3)	P038 (*n* = 2) and P080 (*n* = 1)	P080 (*n* = 2)	–	P038 (*n* = 1)	–
G	*bla*_CTX-M-55_ (*n* = 26)	P046 (*n* = 5)	P046 (*n* = 1), P086 (*n* = 1), and NT (*n* = 2)	P026 (*n* = 1), P089 (*n* = 1), P108 (*n* = 1), P114 (*n* = 1), and NT (*n* = 1)	P026 (*n* = 1), P089 (*n* = 2), P117 (*n* = 1), and NT (*n* = 1)	P010 (*n* = 1), P046 (*n* = 1), P105 (*n* = 1), and NT (*n* = 1)	P010 (*n* = 1), P114 (*n* = 1), and NT (*n* = 1)	–
H	*bla*_CTX-M-55_ (*n* = 17)	P038 (*n* = 1) and P040 (*n* = 1)	P054 (*n* = 3), P125 (*n* = 1), and NT (*n* = 1)	P038 (*n* = 2), P041 (*n* = 2), and P115 (*n* = 1)	P054 (*n* = 3)	P054 (*n* = 1)	P041 (*n* = 1)	–
I	*bla*_CTX-M-55_ (*n* = 6)	P069 (*n* = 1)	P038 (*n* = 1) and P116 (*n* = 2)	P021 (*n* = 1)	–	–	P039 (*n* = 1)	–
	*bla*_CTX-M-65_ (*n* = 15)	P028 (*n* = 1), P031 (*n* = 1), P039 (*n* = 1), and P090 (*n* = 1)	P073 (*n* = 1) and P076 (*n* = 1)	P057 (*n* = 1) and P128 (*n* = 1)	P003 (*n* = 1), P030 (*n* = 1), and P091 (*n* = 1)	P030 (*n* = 2) and P034 (*n* = 1)	P083 (*n* = 1)	–
	*bla*_CMY-2_ (*n* = 2)	–	–	P050 (*n* = 1) and P052 (*n* = 1)	–	–	–	–
J	*bla*_CTX-M-55_ (*n* = 3)	–	P118 (*n* = 2) and P119 (*n* = 1)	–	–	–	–	–
L	*bla*_CTX-M-14_ (*n* = 2)	–	–	P053 (*n* = 1) and P112 (*n* = 1)	–	–	–	–
	*bla*_CTX-M-55_ (*n* = 6)	P106 (*n* = 1)	–	P106 (*n* = 2)	P106 (*n* = 1)	P106 (*n* = 1)	P106 (*n* = 1)	–
	*bla*_CMY-2_ (*n* = 2)	–	–	–	P060 (*n* = 2)	–	–	–
M	*bla*_CTX-M-55_ (*n* = 8)	P004 (*n* = 1)	P029 (*n* = 1), P035 (*n* = 1), P078 (*n* = 1), and P104 (*n* = 2)	P104 (*n* = 1)	P024 (*n* = 1)	–	–	–
N	*bla*_CTX-M-55_ (*n* = 8)	P106 (*n* = 3)	–	P103 (*n* = 1)	P005 (*n* = 1), P102 (*n* = 2), and P126 (*n* = 1)	–	–	–
O	*bla*_CTX-M-15_ (*n* = 2)	–	–	–	NT (*n* = 1)	P059 (*n* = 1)	–	–
	*bla*_CTX-M-55_ (*n* = 8)	P067 (*n* = 2)	P124 (*n* = 1)	P010 (*n* = 1)	P067 (*n* = 1)	P068 (*n* = 1) and P124 (*n* = 1)	P068 (*n* = 1)	–
	*bla*_CMY-2_ (*n* = 4)	P049 (*n* = 1) and P062 (*n* = 1)	–	–	P071 (*n* = 1)	P056 (*n* = 1)	–	–
P	*bla*_CTX-M-55_ (*n* = 13)	P020 (*n* = 2) and P099 (*n* = 1)	P047 (*n* = 1)	P009 (*n* = 2) and P018 (*n* = 1)	P025 (*n* = 1)	P009 (*n* = 1) and P047 (*n* = 3)	P047 (*n* = 1)	–
Q	*bla*_CTX-M-55_ (*n* = 17)	P001 (*n* = 1), P098 (*n* = 3), and NT (*n* = 1)	P002 (*n* = 1), P006 (*n* = 1), P098 (*n* = 1), and NT (*n* = 2)	P016 (*n* = 1)	P058 (*n* = 1)	P006 (*n* = 1), P098 (*n* = 1), and NT (*n* = 1)	P070 (*n* = 1) and P098 (*n* = 1)	–
R	*bla*_CTX-M-14_ (*n* = 10)	–	P061 (*n* = 3)	P061 (*n* = 4)	–	P061 (*n* = 2)	P061 (*n* = 1)	–
	*bla*_CTX-M-15_ (*n* = 7)	P087 (*n* = 4)	–	–	P087 (*n* = 1) and P088 (*n* = 2)	–	–	–
	*bla*_CTX-M-65_ (*n* = 1)	–	–	–	P051 (*n* = 1)	–	–	–
	*bla*_CMY-2_ (*n* = 1)	–	–	–	–	P072 (*n* = 1)	–	–
S	*bla*_CTX-M-14_ (*n* = 8)	P015 (*n* = 1)	–	–	P033 (*n* = 2) and P077 (*n* = 1)	P055 (*n* = 1) and P075 (*n* = 1)	P017 (*n* = 1) and P055 (*n* = 1)	–
	*bla*_CTX-M-15_ (*n* = 2)	–	P074 (*n* = 1)	P074 (*n* = 1)	–	–	–	–
	*bla*_CTX-M-27_ (*n* = 2)	P064 (*n* = 1)	P019 (*n* = 1)	–	–	–	–	–
	*bla*_CTX-M-55_ (*n* = 4)	–	–	P012 (*n* = 1), P036 (*n* = 1), and P037 (*n* = 1)	P082 (*n* = 1)	–	–	–
	*bla*_CTX-M-65_ (*n* = 2)	–	–	–	–	P079 (*n* = 1) and P113 (*n* = 1)	–	–
	*bla*_CMY-2_ (*n* = 2)	P027 (*n* = 1) and P063 (*n* = 1)	–	–	–	–	–	–

^*a*^ Underlining indicates the PFGE types detected in the same farm.

^*b*^ NT, not tested.

^*c*^ –, not detected.

In most farms, the same PFGE patterns were observed within the same age group. Among the 18 farms, identical patterns were confirmed in 11 farms for weaned pigs, 11 for growing pigs, 7 for finishing pigs, and 12 for sows in ceftiofur-use and ceftiofur non-use farms. Identical PFGE patterns between pigs and their environment were observed in nine farms. Additionally, identical PFGE patterns were found in seven farms between isolates from the floor and ventilation ducts. Notably, in farm A, the same PFGE pattern was detected in both the isolates from drinking water and the growing pigs.

To detect the sequence type (ST) in ESBL/AmpC-harboring *E. coli*, we selected one isolate from each PFGE type. A total of 52 STs were identified in 125 different PFGE patterns (Table 6). Among them, the most frequently observed types were ST457 (*n* = 12), ST410 (*n* = 10), ST101 (*n* = 9), ST75 (*n* = 9), and ST10 (*n* = 8). These five STs comprised 38.4% (48/125) of the isolates. Moreover, the ST131 (*n* = 4) in *bla*_CTX-M-55_ and ST162 (*n* = 1) in *bla*_CTX-M-65_-producing *E. coli* were also detected. Interestingly, seven co-carrying *bla*_CTX-M-55_ and *mcr-1 E. coli* were assigned ST88 (*n* = 6) and ST224 (*n* = 1).

**TABLE 6:** Multilocus sequence typing profiles of ESBL/AmpC-carrying *E. coli* isolated from pigs and their farm environment during 2022–2023 in South Korea

*bla* types	No. of isolates	Distribution of STs (no. of isolates)
*bla*_CTX-M-55_	68	ST457 (*n* = 11), ST75 (*n* = 9), ST101 (*n* = 7), ST410 (*n* = 6), ST131 (*n* = 4), ST88 (*n* = 3), ST2628 (*n* = 3), ST10 (*n* = 2), ST215 (*n* = 2), ST3285 (*n* = 2), ST4450 (*n* = 2), ST48 (*n* = 1), ST58 (*n* = 1), ST224 (*n* = 1), ST278 (*n* = 1), ST442 (*n* = 1), ST542 (*n* = 1), ST641 (*n* = 1), ST737 (*n* = 1), ST772 (*n* = 1), ST795 (*n* = 1), ST1081 (*n* = 1), ST1244 (*n* = 1), ST1249 (*n* = 1), ST1716 (*n* = 1), ST5229 (*n* = 1), ST6778 (*n* = 1), and ST14922 (*n* = 1)
*bla*_CTX-M-65_	15	ST10 (*n* = 4), ST20 (*n* = 1), ST162 (*n* = 1), ST410 (*n* = 1), ST641 (*n* = 1), ST683 (*n* = 1), ST772 (*n* = 1), ST3339 (*n* = 1), ST3494 (*n* = 1), ST3519 (*n* = 1), ST4450 (*n* = 1), and ST5229 (*n* = 1)
*bla*_CTX-M-1_	10	ST5229 (*n* = 3), ST196 (*n* = 2), ST10 (*n* = 1), ST101 (*n* = 1), ST345 (*n* = 1), ST3326 (*n* = 1), and ST4014 (*n* = 1)
*bla*_CTX-M-14_	9	ST410 (*n* = 2), ST20 (*n* = 1), ST48 (*n* = 1), ST88 (*n* = 1), ST178 (*n* = 1), ST301 (*n* = 1), ST1244 (*n* = 1), and ST4546 (*n* = 1)
*bla*_CTX-M-15_	5	ST1408 (*n* = 2), ST162 (*n* = 1), ST4981 (*n* = 1), and ST10638 (*n* = 1)
*bla*_CTX-M-27_	3	ST410 (*n* = 1), ST1112 (*n* = 1), and ST10638 (*n* = 1)
*bla*_CMY-2_	13	ST10 (*n* = 1), ST12 (*n* = 1), ST93 (*n* = 1), ST101 (*n* = 1), ST345 (*n* = 1), ST453 (*n* = 1), ST457 (*n* = 1), ST641 (*n* = 1), ST761 (*n* = 1), ST2025 (*n* = 1), ST2608 (*n* = 1), ST4417 (*n* = 1), and ST5229 (*n* = 1)

## DISCUSSION

In this study, a significant prevalence of ceftiofur-resistant *E. coli* was found in pigs and their farm environment in both ceftiofur-use and ceftiofur-non-use farms. *bla*_CTX-M-55_ (about 60%) was the main ESBL/AmpC β-lactamase-encoding gene contributing to the horizontal and clonal spread of third-generation cephalosporin resistance in the Korean pig industry.

The emergence of third-generation cephalosporin resistance in Enterobacterales, including *E. coli*, poses a critical danger to humans and animals. We detected ceftiofur resistance in 46% of the *E. coli* isolates, with 61% recovered from pigs and 22.9% from their farm environments. Consistent with this study, a high level of extended-spectrum cephalosporin resistance was found in *E. coli* isolates recovered from pigs and pig farm environments in Korea (21), Taiwan (23), and Cuba (2). In this study, ceftiofur-resistant *E. coli* isolates were selected using ceftiofur-containing agar. The ceftiofur resistance determining method used in this investigation might affect the high prevalence of ceftiofur-resistant *E. coli*.

We found that the prevalence of ceftiofur-resistant *E. coli* was significantly higher in ceftiofur-use farms than in non-use farms. The development of antimicrobial resistance is caused by the excessive and improper use of antimicrobial agents for prevention and treatment, including third-generation cephalosporin, which is used formally in veterinary applications to treat diverse gram-negative bacterial infections (24).

Cephalosporin resistance usually results from bacterial evolution in response to the use of antimicrobials (25), triggering a steady rise in the prevalence of third-generation cephalosporin-resistant *E. coli* globally, including in Korea (26–28). Ceftiofur is approved for the treatment of enteric and respiratory diseases in pigs in Korea, and regulation of this tendency is required to control the resistance of third-generation cephalosporin. Interestingly, previous reports showed that third-generation cephalosporin-resistant *E. coli* is also frequently detected in the ceftiofur-no-use pig farms worldwide (29–31), which is consistent with our investigation.

Third-generation cephalosporins are frequently used for the management and treatment of post-weaning diarrhea, endotoxin shock, and edema, which are the main problems facing the swine industry (32). Moreover, the frequent use of third-generation cephalosporins in the growing, finishing, and sows also contributed to the significant emergence of *E. coli* resistance in these stages of swine production (21). Similarly, *E. coli* isolates from pig farm environments demonstrated significant resistance to third-generation cephalosporin, as reported in Thailand (11), Ireland (29), and Korea (33). In addition, third-generation cephalosporin-resistant *E. coli* also demonstrated resistance to other cephalosporins (31). In our study, it was noticeable that in both pig and farm environment isolates, the rate of ceftiofur resistance was significantly higher than that of the ceftiofur-non-use isolates, consistent with the previously published reports (34, 35).

The findings indicate that while ceftiofur non-use farms did not administer ceftiofur, they still used other classes of antimicrobials, such as aminoglycosides and tetracyclines, particularly in weaner and grower pigs. Notably, the use of these antimicrobials is more common in the weaner and grower age groups compared to other age groups, such as finishers and sows. This practice might contribute to the co-selection of ceftiofur resistance. Our investigation revealed that most transconjugants exhibited resistance not only to ceftiofur but also to other commonly used antimicrobials in pigs. Furthermore, ceftiofur-resistant *E. coli* may be transmitted between the environment and pigs. Identical PFGE patterns were observed in weaners, growers, and the surrounding environment, with farms showing matched PFGE patterns in weaners including A, B, F, and Q and in growers including A, B, F, H, O, P, and Q. Moreover, we observed that the isolates from the finisher group of the ceftiofur-use farm (90%) showed the highest level of ceftiofur resistance, which is unusual in this study. The reasons for this high prevalence of ceftiofur resistance in finisher pigs in ceftiofur-use farms might be due to clonal spread in the farms among pigs and/or pigs and the farm environment. Moreover, among five ceftiofur-use farms, identical PFGE patterns from finisher pigs and the environment were observed in two farms (G and L). In addition, identical PFGE patterns in finisher pigs and other age groups were observed in three farms (G, L, and S).

In the farm environment, most ceftiofur-resistant *E. coli* isolates were obtained from the floor (50.7%) and ventilation surface (22.5%). Consistent with the previous investigations, the prevalence of extended-spectrum cephalosporin-resistant *E. coli* in this study was also detected in farm environments, including floors and ventilations (29). This might be due to the shedding of resistant bacteria in these farm facilities, providing evidence that not only pigs but also their living environment are potential sources of antimicrobial-resistant bacteria (21).

In this investigation, *bla*_CTX-M-55_ was mostly detected in pigs (59.3%) and the environment (62.3%). Once *bla*_CTX-M-55_ was a less prevalent gene found in *Enterobacteriaceae* from humans and food animals; however, recently, there has been a growing occurrence in *E. coli* isolates from humans and pigs in Europe (36), Asia (23, 37, 38), and North America (39), indicating the potential for a global epidemiological change in the distribution of this ESBL type. The predominant prevalence of *bla*_CTX-M-55_ may be due to its widespread distribution capabilities in *E. coli*, which are facilitated by the presence of different plasmids, especially IncI1 (40). Furthermore, 11.1% and 10.2% of the pig *E. coli* isolates possessed *bla*_CMY-2_ and *bla*_DHA1_, respectively. These genes were frequently identified in the third-generation cephalosporin-resistant *E. coli* obtained from humans and pigs (41, 42). Moreover, the co-occurrence of *bla*_CTX-M_ with the AmpC genes *bla*_DHA_ and *bla*_CMY-2_ was found in *E. coli* strains consistent with the previous investigation (11). These findings emphasize the need for coordinated control of ESBL- and AmpC β-lactamase-harboring *E. coli* in humans and other animals.

The present study revealed that 38.7% (104/269) of harboring ESBL/AmpC β-lactamase *E. coli* exhibited the ability to transfer the β-lactamase genes. Differences in transferability were observed among *bla*_CTX-M_ types, where *bla*_CTX-M-1_ was highly transferred (85.7%), followed by *bla*_CTX-M-55_ (46.6%) and *bla*_CTX-M-14_ (40%) to the recipient *E. coli*. A previous investigation showed that 25% of the ESBL-producing *E. coli* isolated from pigs and their farm environment were able to be transferred to the recipient (17). Moreover, previous studies showed that *bla*_CTX-M-1_, *bla*_CTX-M-55_, and/or *bla*_CTX-M-14_ are also among the highly transferred ESBL-carrying *E. coli* strains recovered from food animals including pigs (17, 21). It was also shown that isolates possessing identical genes to the donor isolates indicate that *E. coli* producing β-lactamase can be spread clonally to humans or other animals through direct contact or with pig-origin contaminated food products (17, 20). Thus, our outcome exhibits that ESBL-carrying *E. coli* strains have the potential to be extensively disseminated to pigs and their farm environment and contribute to the transmission of these genes to humans directly or indirectly.

In our study, the transconjugant of *bla*_CTX-M-55_-carrying isolates contains IncFIB (85.3%), and the *bla*_CTX-M-1_-harboring isolate contains IncI1 (91.7%). The previous investigation showed the frequent existence of the IncFIB plasmid in *E. coli* strains isolated from humans and pigs (11). In addition, the presence of IncI1 in *E. coli* isolates from humans and food animals was also confirmed in the previous study (43). The IncFIB and IncI1 conjugative plasmids play a crucial role in the rapid and extensive spread of ESBL-carrying *E. coli* (11).

Mobile genetic elements are essential for accumulating, interacting, and maintaining antimicrobial resistance (44). They facilitate the mobilization and dispersion of antimicrobial resistance genes, contributing to the spread of resistance (45). In this investigation, most of the transposable genetic elements were identified in two predominantly detected genetic structures, types I and II of the five types (I–V). Among them, the transposable elements IS*26*-IS*Ecp1-bla*_CTX-M-55_-*orf477* (76%) and IS*Ecp1-bla*_CTX-M-55_-*orf477* (22.7%) were the most prevalent in type I, while *bla*_CTX-M-65_-IS*903* (50%) in type II and *bla*_CTX-M-14_-IS*903* (100%) were the most commonly detected in type IV genetic structure. It was demonstrated that the transposable elements IS*Ecp1* and *orf477*, situated downstream, play a significant role in activating and spreading *bla*_CTX-M_ genes (46). Moreover, the transposable unit IS*903* can facilitate the transfer of *bla*_CTX-M_ genes across transmissible plasmids in bacteria (47).

We have suggested that various antimicrobial resistance patterns were transferred along with *bla*_CTX-M_. Of them, chloramphenicol, tetracycline, and aminoglycoside resistance patterns were frequently observed in association with *bla*_CTX-M-55_, *bla*_CTX-M-1_, *bla*_CTX-M-14_, and *bla*_CTX-M-15_, while all *bla*_CTX-M-55_-carrying transconjugants transferred one more antimicrobial with tetracycline being common. The previous investigation demonstrated that resistance to these antimicrobials was revealed to be transferred with *bla*_CTX-M_ in *E. coli* isolated from pigs (17, 28). Moreover, *bla*_CTX-M-55_-carrying *E. coli* transferring with tetracycline resistance was found in isolates from food animals (48). In addition, it was shown that the *bla*_CTX-M_ gene in *E. coli* can be transmitted horizontally along with the *floR* gene (which confers resistance to chloramphenicol), the *tetA* gene (which confers resistance to tetracycline), and the *aac(3)-IV* gene (which confers resistance to aminoglycoside), contributing to the elevated level of resistance to these antimicrobials (49). The *bla*_CTX-M_ was co-selected with the use of other antimicrobials such as chloramphenicol, gentamicin, and tetracycline. These results suggested that ceftiofur-resistant *E. coli* isolates in non-use farms are also highly prevalent.

The PFGE and multilocus sequence typing (MLST) analyses are commonly used to examine genetic relatedness and global epidemiology of the isolates (50). In this study, we found that ESBL/AmpC-harboring *E. coli* strains revealed various PFGE patterns, suggesting that they emerged from distinct clones. Nevertheless, identical patterns were detected among pigs within the same farm and their farm environment. In addition, similar PFGE patterns were identified in most farms within the same age range. Identical PFGE patterns in different pigs and their environment suggest the transmission of ESBL/AmpC-harboring *E. coli*, consistent with the previous investigation (51). Moreover, it was demonstrated that ESBL/AmpC-producing *E. coli* is widely distributed by clonal transmission in humans and animals worldwide (11, 52–54). The high prevalence of ceftiofur resistance in pigs in ceftiofur non-use farms might be due to their transmission among pigs and their environment. This result suggests the importance of cleaning and disinfection to control antimicrobial resistance in pig farms.

Based on MLST analysis, ST457 was the predominant ST in pigs and their farm environment, followed by ST410, ST101, ST75, and ST10. The ST457 is a frequently detected ST in *E. coli* isolated from both pigs and humans (55). Moreover, ST410, ST101, ST75, and ST10 were found to be associated with ESBL/AmpC β-lactamase-encoding genes detected in *E. coli* from pigs and humans (21, 56). In addition, we found that STs were different based on *bla*_CTX-M_ types. The most predominant STs of the *bla*_CTX-M-55_, *bla*_CTX-M-65_, *bla*_CTX-M-1_, and *bla*_CTX-M-14_ were ST457, ST10, ST5229, and ST410, respectively, consistent with previous studies showing that STs of each *bla*_CTX-M_ type were different (57). It was revealed that STs in different *bla*_CTX-M_ types were detected in *E. coli* isolated from pigs (58, 59). Nevertheless, they also have the capability to infect humans, presenting severe health risks, consistent with previous investigations that have shown *E. coli* harboring antibiotic resistance showed similarity with STs (60).

In this study, differences in antibiotic resistance were observed based on the ESBL and/or AmpC-type genes. Among them, amoxicillin/clavulanic acid showed high resistance in *bla*_CMY-2_ and co-producing *bla*_CTX-M-55_ and *bla*_DHA-1_ *E. coli. E. coli* is often sensitive to amoxicillin-clavulanate; however, resistance to this antibacterial has recently emerged (61, 62). This resistance mechanism generally includes the acquired plasmid-encoded cephalosporinase (AmpC type) and overproduction of the chromosomal AmpC in *E. coli* (63). Moreover, resistance to chloramphenicol, ciprofloxacin, and tetracycline, among the non-beta-lactam antibiotics, was high in all *bla*_CTX-M_ or *bla*_CMY-2_ types and in some of the co-producer *E. coli*. The co-resistance of non-beta-lactam antibiotics and cephalosporin is frequently detected in *E. coli* isolates from humans and food animals (64, 65).

Colistin is one of the last-resort antimicrobials for the treatment of multidrug-resistant bacteria (66). However, the co-presence of the third-generation cephalosporin-resistant gene and colistin creates further complexities for bacterial infection treatment. This is the first study on *E. coli* co-carrying *bla*_CTX-M-55_ and *mcr-1* in food animals in Korea. Moreover, these co-carrying *bla*_CTX-M-55_ and *mcr-1* isolates were associated with ST88 detected in this investigation. Previous studies revealed that the plasmids harboring *bla*_CTX-M_*-*type ESBL/AmpC β-lactamases are frequently present in *mcr-1*-carrying isolates (11). Particularly, the *bla*_CTX-M-55_-carried plasmid contains *mcr-1* in *E. coli* isolates from livestock (67) and humans (68). Plasmids containing numerous resistance genes associated with sequence type are especially problematic, as a single instance of horizontal gene transfer could lead to the bacteria becoming extensive or pan-drug-resistant (69).

This is the first report comparing ceftiofur-resistant *E. coli* between ceftiofur-use and non-use farms in Korea. The prevalence of ceftiofur resistance in *E. coli* was highly detected in both ceftiofur-use and ceftiofur non-use farms. *bla*_CTX-M-55_ was predominantly detected among ESBL/AmpC β-lactamase harboring *E. coli*. Moreover, ST457, ST75, ST101, and ST410 were the most predominantly detected STs associated with *bla*_CTX-M-55_. In addition, most *bla*_CTX-M-55_ genes carried mobile genetic elements IS*ECP1* and IS*26*, contributing to horizontal transmission of resistance. Furthermore, identical PFGE patterns were observed in pigs and their environment. These results suggest that antimicrobial use pressure and horizontal and clonal spread are risk factors for the emergence of ESBL-carrying *E. coli* in the pig industry. Thus, to control antimicrobial resistance, restricting antimicrobial use and implementing hygienic measures are needed in pig farms.

## MATERIALS AND METHODS

### Sample collection

In total, 585 samples (354 from feces and 231 from the farmhouse environment) were obtained from 18 different ceftiofur-use (*n* = 5) and ceftiofur-non-use pig farms (*n* = 13) in Korea between 2022 and 2023. Among them, fecal samples were gathered from four age groups: 90 weaners, 89 growers, 85 finishers, and 90 sows, and environment samples were collected from four sites: 71 from floors, 71 from ventilation, 71 from feed, and 18 from drinking water. Approximately 10 g of feces collected per pig was contained in a sterile plastic tube. Environmental samples were collected by swabbing pen floors, ventilation systems, feed, and drinking water using 10 cm × 10 cm gauze pads wetted with 1% sterile skim milk. The collected samples were placed in tightly secured sterile plastic bags and quickly sent to the laboratory in an ice-cooled container for subsequent analysis.

### Isolation and identification of ceftiofur-resistant *E. coli*

The fecal and farm environmental samples were spread on the MacConkey agar (Becton Dickinson, NV, USA) containing ceftiofur (2 µg/mL) overnight at 37°C. *E. coli* was confirmed by matrix-assisted laser desorption and ionization-time-of-flight spectroscopy (MALDI-TOF; Bruker Corporation, MA, USA). Briefly, the bacterial colonies of each isolate were cultured on tryptic soy agar (Becton Dickinson, NV, USA) for 24 h at 37°C. A small portion of colony biomass was dispersed throughout the MALDI plate’s designated target sites. One microliter of matrix solution containing alpha-cyano-4-hydroxycinnamic acid was added to every sample spot, followed by air-drying to facilitate matrix-sample co-crystallization. The developed MALDI plate was placed into the linear scanning mode of the MALDI-TOF mass spectrometry device. The plate was then read, and the results were correlated with the database representing the unique spectrum of specific bacteria. A single *E. coli* isolate from each sample was chosen for subsequent analysis.

### Antimicrobial susceptibility testing

The broth microdilution technique was used with a commercial Sensititre panel KRNVF (Thermo Fisher, Waltham, USA) to ascertain the MICs of the *E. coli* isolates against the tested antimicrobials (70). *E. coli* ATCC25922 was used as a quality reference isolate.

### Determination of β-lactamase-encoding genes

The PCR amplification was carried out on the ceftiofur-resistant *E. coli* isolates using specific primers for ESBL/AmpC β-lactamase-encoding genes (*bla*_CTX-M_ and *bla*_CMY_). The sequencing of PCR products was accomplished by ABI3730XL DNA sequencing analyzer (SolGent, Daejeon, Korea), and the sequences were validated by comparing with known sequences present in the GenBank database using the Basic Local Alignment Search Tool (BLAST) in the National Center for Biotechnology Information website (https://www.ncbi.nlm.nih.gov/BLAST). The primers and PCR conditions are listed in Table S1.

### Conjugation assay

The conjugation assay was performed to assess the transferability of β-lactamase genes by filter-mating method using rifampin-resistant *E. coli* RG488 as a recipient (71). In brief, fresh-grown bacteria were mated at a 1:10 ratio between donor and recipient and subsequently collected on a membrane filter. The collected bacteria were incubated on tryptic soy agar plates overnight and selected on MacConkey agar plates supplemented with rifampin (100 µg/mL) and ceftiofur (25 µg/mL). Finally, all the transconjugants were assayed for antibiotic susceptibility and detecting β-lactamase genes.

### Molecular characterization of *bla*_CTX-M_ environment

The PCR-based replicon typing of the extracted DNA was performed according to the previously described primers and method (72). Moreover, the genomic environment of the β-lactamase gene was determined by multiplex PCR and Sanger sequencing analysis. The specific primers for the upstream and downstream of the β-lactamase gene (IS*Ecp1*, *orf477*, IS*26*, and IS*903*) were used along with β-lactamase gene primers (Table S1).

### Pulsed-field gel electrophoresis and multilocus sequence typing

The PFGE was performed to determine the genetic similarity of ESBL/AmpC-harboring genomic DNA digested with *Xba*I (Takara Bio, Inc., Shiga, Japan) according to the protocol outlined in the previous study (73). The unweighted pair group linkage with the Dice coefficient was used to determine the similarity of PFGE bands. MLST was accomplished to assay the clonal relationship of ceftiofur-resistant *E. coli* isolates following the previous method (17). The amplification and partial sequencing of the seven housekeeping genes (*adk*, *fumC*, *gyrB*, *icd*, *mdh*, *purA*, and *recA*) were carried out using specific primers. The allele profile and STs for *E. coli* were identified from the web-based MLST database (https://pubmlst.org/databases/).

### Statistical analysis

The obtained data were analyzed using Rex Software (version 3.0.3; RexSoft Inc., Seoul, Korea), encompassing the *χ*^2^ test. A *P*-value of less than 0.05 was deemed statistically significant.

## Data Availability

The data produced in this study are included within the article and its supplemental material. Further correspondence and requests should be addressed to the corresponding author.

## SUPPLEMENTAL MATERIAL

Fig. S1 aem.02525-24-s0001.docx Percentage of *bla* gene distribution (pig fecal samples/environmental samples) among different farms located in various provinces in South Korea.

Table S1 aem.02525-24-s0002.docx Primer sequences and PCR conditions.

ASM does not own the copyrights to Supplemental Material that may be linked to, or accessed through, an article. The authors have granted ASM a non-exclusive, world-wide license to publish the Supplemental Material files. Please contact the corresponding author directly for reuse.
