# Supplementary material for: High prevalence of blaCTX-M-55-carrying Escherichia coli in both ceftiofur-use and non-use pig farms
Source: Appl Environ Microbiol. 2025 Jul 15;91(8):e02525-24. doi: 10.1128/aem.02525-24 (PMC12366300; doi:10.1128/aem.02525-24)

Fig. S1. *bla* gene distribution % (pig fecal samples/environmental samples) among different farms located in various provinces in South Korea.
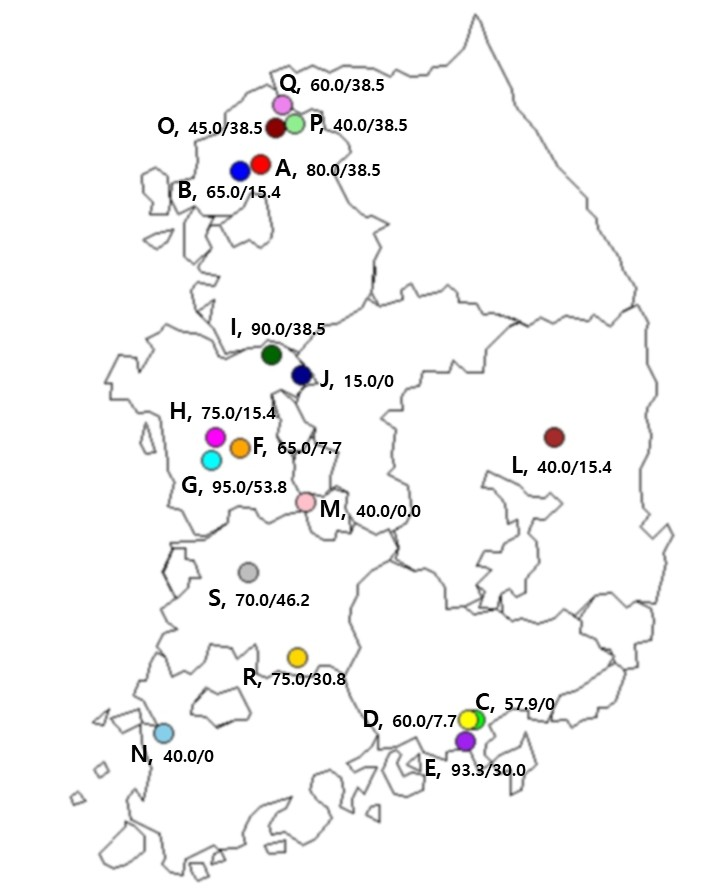

Supplement: Fig. S1 — Percentage of bla gene distribution (pig fecal samples/environmental samples) among different farms located in various provinces in South Korea. [file aem.02525-24-s0001.docx]
