# Supplementary material for: High prevalence of blaCTX-M-55-carrying Escherichia coli in both ceftiofur-use and non-use pig farms
Source: Appl Environ Microbiol. 2025 Jul 15;91(8):e02525-24. doi: 10.1128/aem.02525-24 (PMC12366300; doi:10.1128/aem.02525-24)
Supplement: Table S1 — Primer sequences and PCR conditions. [file aem.02525-24-s0002.docx]

Table S1. List of primer sequences and polymerase chain reaction (PCR) conditions

| Primer | Sequence (5'-3') | Ampliconsize (bp) | PCR condition | References |
| --- | --- | --- | --- | --- |
| CTX-M-Universal^a^ | ATGTGCAGYACCAGTAARGTKATGGC | 593 | 35 cycles; 95℃ 1min + 60℃ 1min + 72℃ 1min | (1) |
|  | TGGGTRAARTARGTSACCAGAAYCAGCGG |  |  |  |
| CTX-M-1 families^a^ | AAGACTGGGTGTGGCATTGA | 670 | 35 cycles; 95℃ 1min + 60℃ 1min + 72℃ 1min | (1) |
|  | AGGCTGGGTGAAGTAAGTGA |  |  |  |
| CTX-M-2 families^a^ | CTGGAAGCCCTGGAGAAAAG | 789 | 35 cycles; 95℃ 1min + 60℃ 1min + 72℃ 1min | (1) |
|  | TACCTCGCTCCATTTATTGC |  |  |  |
| CTX-M-8 families^a^ | GCCTGTATTTCGCTGTTG | 686 | 35 cycles; 95℃ 1min + 60℃ 1min + 72℃ 1min | (1) |
|  | TGTCATTCGTCGTACCATAA |  |  |  |
| CTX-M-9 families^a^ | GCTTTATGCGCAGACGAGTG | 703 | 35 cycles; 95℃ 1min + 60℃ 1min + 72℃ 1min | (1) |
|  | GCCAGATCACCGCAATATCA |  |  |  |
| CTX-M-1 sequencing | CAGCACTTTTGCCGTCTAAG | 1130 | 35 cycles; 94℃ 30 sec + 55℃ 30 sec + 72℃ 1min | (2) |
|  | AAAAATGATTGAAAGGTGGT |  |  |  |
| CTX-M-9 sequencing | GAAGCAGTCTAAATTCTTCGTGAAATAG | 1100 | 35 cycles; 94℃ 30 sec + 60℃ 30 sec + 72℃ 1min | (2) |
|  | GGGCCAGTTGGTGATTTTGA |  |  |  |
| *MOX-1, MOX-2, CMY-1, CMY-8 to CMY-11^b^* | GGGCCAGTTGGTGATTTGA | 520 | 25 cycles; 94℃ 30 sec + 64℃ 30 sec + 72℃ 1min | (3) |
|  | GCTGCTCAAGGAGCACAGGAT |  |  |  |
| *LAT-1 to LAT-4, CMY-2 to CMY-7, BIL-1^b^* | TGGCCAGAACTGACAGGCAAA | 462 | 25 cycles; 94℃ 30 sec + 64℃ 30 sec + 72℃ 1min | (3) |
|  | TTTCTCCTGAACGTGGCTGGC |  |  |  |
| *DHA-1, DHA-2^b^* | AACTTTCACAGGTGTGCTGGGT | 405 | 25 cycles; 94℃ 30 sec + 64℃ 30 sec + 72℃ 1min | (3) |
|  | CCGTACGCATACTGGCTTTGC |  |  |  |
| *ACC^b^* | AACAGCCTCAGCAGCCGGTTA | 346 | 25 cycles; 94℃ 30 sec + 64℃ 30 sec + 72℃ 1min | (3) |
|  | TTCGCCGCAATCATCCCTAGC |  |  |  |
| *MIR-1T, ACT-1^b^* | TCGGTAAAGCCGATGTTGCGG | 302 | 25 cycles; 94℃ 30 sec + 64℃ 30 sec + 72℃ 1min | (3) |
|  | CTTCCACTGCGGCTGCCAGTT |  |  |  |
| *FOX-1 to FOX-5^b^* | AACATGGGGTATCAGGGAGATG | 190 | 25 cycles; 94℃ 30 sec + 64℃ 30 sec + 72℃ 1min | (3) |
|  | CAAAGCGCGTAACCGCATTGG |  |  |  |
| *ISEcp1^c^* | CGAGAGAAATTACACCGGTCA | 1547 | 35 cycles; 95℃ 30sec + 53℃ 30sec + 72℃ 1min | (4) |
|  | AGCCCTTCAATGCTGATGTC |  |  |  |
| *orf477 ^c^* | TGTATGCGATGTCTGAACTG | 1238 | 35 cycles; 95℃ 30sec + 50℃ 30sec + 72℃ 1min | (5) |
|  | CTCAATAGCAGCTCGGAATA |  |  |  |
| *IS903 ^c^* | TACCGAGCTGTTCCTTGTGG | 836 | 35 cycles; 95℃ 30sec + 53℃ 30sec + 72℃ 1min | (6) |
|  | CGGTTGTAATCTGTTGTCCA |  |  |  |
| *IS26 ^c^* | CTGCTTTACCAACAACATTCC | 618 | 35 cycles; 95℃ 30sec + 56℃ 30sec + 72℃ 1min | (4) |
|  | TTGTCCGGGTTGTACTCGTC |  |  |  |

^a^ Primer sequences used in *bla*_CTX-M,_

^b^ Primer sequences used in *bla*_CMY_

^c^ Primer sequences used in determinantion of genetic environment of *bla*_CTX-M_ and *bla*_CMY-2_

**References**

1. Batchelor M, Threlfall EJ, Liebana E. 2005. Cephalosporin resistance among animal-associated Enterobacteria: a current perspective. Expert Rev Anti Infect Ther 3:403–417. https://doi.org/10.1586/14787210.3.3.403

2. Yaita K, Aoki K, Suzuki T, Nakaharai K, Yoshimura Y, Harada S, Ishii Y, Tachikawa N. 2014. Epidemiology of extended-spectrum β-lactamase producing *Escherichia coli* in the stools of returning Japanese travelers, and the risk factors for colonization. PLoS One 9:e98000. https://doi.org/10.1371/journal.pone.0098000

3. Pérez-Pérez FJ, Hanson ND. 2002. Detection of plasmid-mediated AmpC β-lactamase genes in clinical isolates by using multiplex PCR. J Clin Microbiol 40:2153–2162. https://doi.org/10.1128/jcm.40.6.2153-2162.2002

4. Eckert C, Gautier V, Arlet G. 2006. DNA sequence analysis of the genetic environment of various *bla* _CTX-M_ genes. J Antimicrob Chemother 57:14–23. https://doi.org/10.1093/jac/dki398

5. Saladin M, Cao VTB, Lambert T, Donay J-L, Herrmann J-L, Ould-Hocine Z, Verdet C, Delisle F, Philippon A, Arlet G. 2002. Diversity of CTX-M β-lactamases and their promoter regions from *Enterobacteriaceae* isolated in three Parisian hospitals. FEMS Microbiol Lett 209:161–168. https://doi.org/10.1111/j.1574-6968.2002.tb11126.x

6. Tamang MD, Gurung M, Kang M-S, Nam H-M, Moon DC, Jang G-C, Jung S-C, Park YH, Lim S-K. 2014. Characterization of plasmids encoding CTX-M β-lactamase and their addiction systems in *Escherichia coli* isolates from animals. Vet Microbiol 174:456–462. https://doi.org/10.1016/j.vetmic.2014.10.004
